# ROS-Sensitive Nanoparticles Co-delivering Dexamethasone and CDMP-1 for the Treatment of Osteoarthritis Through Chondrogenic Differentiation Induction and Inflammation Inhibition

**DOI:** 10.3389/fbioe.2021.608150

**Published:** 2021-01-28

**Authors:** Xiaodong Wu, Pengpeng Li, Jian Cheng, Qiang Xu, Beiji Lu, Conghui Han, Weiling Huo

**Affiliations:** ^1^Department of Orthopaedics, Xuzhou Central Hospital, Xuzhou, China; ^2^Xuzhou Clinical School of Xuzhou Medical University, Xuzhou, China; ^3^Bengbu Medical College, Bengbu, China

**Keywords:** osteoarthritis (OA), reactive oxygen species (ROS), nanomicelle, dexamethasone, cartilage-derivedmor-phogeneticprotein-1 (CDMP-1)

## Abstract

**Objective:** Osteoarthritis (OA) is a common subtype of arthritis. To date, treatment of OA focuses primarily on alleviating pain and improving joint function. The lack of a vascular system within synovial joints and the rapid removal of agents due to synovial exchange hinder continuous delivery of OA drugs. However, these obstacles are being addressed by promising nanoscale drugs.

**Methods:** We synthesize and assemble a hydrogen peroxide [H_2_O_2_, belongs to the category of active oxygen species (ROS)]-sensitive nanomicelle, which is loaded with the anti-inflammation drug dexamethasone and chondrogenic differentiation factor cartilage-derivedmor-phogeneticprotein-1. The micelle can induce bone marrow mesenchymal stem cells to repair cartilage while inhibiting joint inflammation.

**Results:** The prepared nanoparticles were of uniform size and displayed an obvious core-shell structure. Under H_2_O_2_ stimulation, the shell layer could be removed gradually. The drug-loaded micelle effectively inhibited proliferation of activated macrophages, induced macrophage apoptosis with an anti-inflammatory effect, and caused the BMSCs to differentiate into chondrocytes.

**Conclusion:** This work provides an experimental and theoretical basis for further development of a drug-loaded micelle in the healing of osteoarthritis.

## Introduction

Osteoarthritis (OA), a degenerative form of arthritis, causes articular cartilage damage and affects entire joints (Miller et al., 2019; Hunt et al., 2020). Because cartilage is a non-vascular, non-lymphoid, and non-nerve tissue (Zhang et al., 2019a,b) and chondrocytes are highly differentiated cells with little potential for proliferation and migration, self-repair and regeneration of cartilage is limited (Roseti et al., 2019; Zhu et al., 2020).

Several surgical procedures have been operated to repair cartilage, including micro fractures (Bergink et al., 2019), mosaics (Jordan et al., 2017) and autologous chondrocytes (Stoop et al., 2007), but long-term success has proved elusive. To meet clinical needs and achieve long-term efficacy, non-invasive therapies that promote cartilage regeneration and exert anti-inflammatory effects are being designed (Boulocher et al., 2007; Palmer et al., 2013). At present, treatment of OA focuses primarily on alleviating pain and improving joint function (Zhu et al., 2020). Glucocorticoids (Cooper et al., 2016; Zhou et al., 2019) are often used to relieve joint pain during inflammation, but they are only effective in the short term. Hyaluronic acid drugs are able to relieve the symptoms of various types of osteoarthritis, but they tend to rapidly exit the joint cavity and provide relief for no more than 1–3 months.

The lack of a vascular system within the synovial joint (Baboolal et al., 2016; Watt et al., 2020) impedes the delivery of potent molecules to target sites during systemic administration. The rapid removal of locally delivered therapeutic agents due to synovial exchange poses another challenge (Jia et al., 2011). The development of nanotechnology provides a promising alternative direction for the development of drug delivery systems for OA (Yang et al., 2017; Zhao et al., 2021).

Nanoparticle drugs offer several advantages over traditional drugs, including increased drug hydrophilicity, reduced side effects and increased circulation time *in vivo* (Zhu et al., 2020). Various functions can work through modification, such as clearance of reactive oxygen species (ROS), lesion-site imaging, photo-dynamic therapy, multi-drug-loading and targeting of lesion sites.

Recent studies have pointed out that the loss of cartilage matrix is closely related to oxidative stress. It is generally recognized that when reactive oxygen species (ROS) are overexpressed, the function of articular cartilage is degraded (Crivelli et al., 2019; Wegner et al., 2019; Shin et al., 2020). By introducing ROS responsive structural components (-SeSe -) into nanoparticles, it provides a basis for targeting the construction of nano-drugs for precision therapy.

Dexamethasone (DEX) is widely used in the treatment of various inflammatory diseases. It can inhibit the accumulation of inflammatory cells, including macrophages and white blood cells, and inhibit the phagocytosis, the release of lysosomal enzymes and the synthesis and release of inflammatory chemical intermediaries. DEX is mainly used in the treatment of OA to eliminate inflammatory response and relieve pain in inflammatory sites (Said Ahmed et al., 2019; Zhao et al., 2019).

Osteoarthritis is fundamentally degenerative arthritis caused by the injury of articular cartilage (Brown et al., 2020). Therefore, the key to the treatment of OA lies in the repair of damaged articular cartilage (Cui et al., 2019). With the progress of materials and biological science, biomaterials have great application prospect in cartilage repair and bone repair (Zhu et al., 2020). The combination of bioactive factors and engineered 3D scaffolds can limit the decrease of biological activity, reduce the occurrence of complications and achieve targeted therapy (Wang et al., 2019; Cui et al., 2020).

Cartilage-derive morphogenetic protein 1 (CDMP-1), as a cytokine which has the ability to induce the proliferation and differentiation of osteoblasts, promote the differentiation of bone marrow mesenchymal stem cells into chondrocytes for repair(Dobbs et al., 2005).

**Scheme 1:**
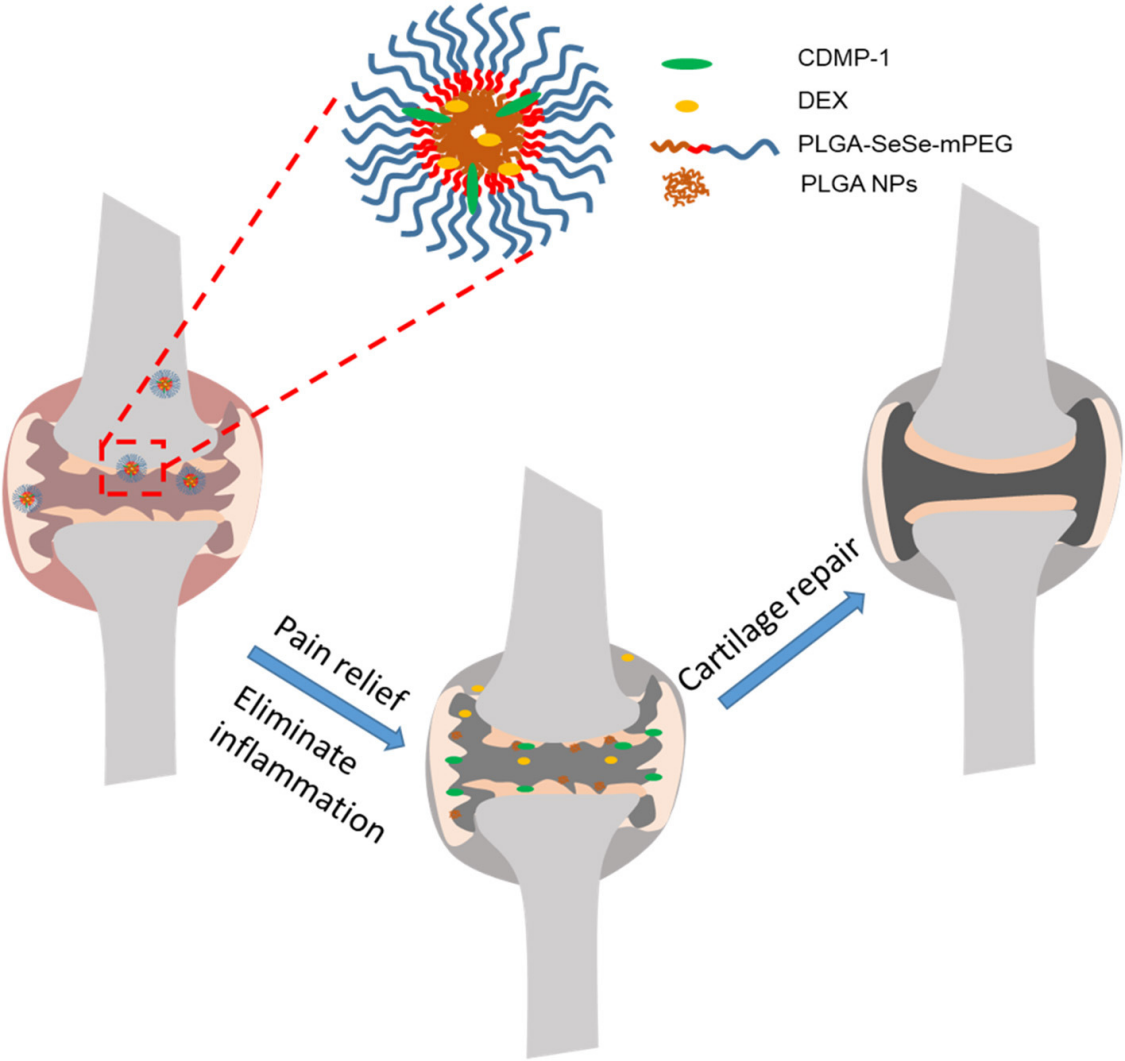
DLNPs are injected into the joint cavity of OA, and the high concentration of ROS in the articular cavity leads to the fracture of –SeSe-. The slow release of DEX reduces both pain and inflammation. The aggregated PLGA particles distributed on the surface of damaged cartilage serve as cartilage scaffoldings, and CDMP-1 biological factors in PLGA particles further induce BMSCs to transform into cartilage and repair damaged cartilage tissues. PLGA, poly(lactic-co-glycolic acid; DEX, dexamethasone; CDMP-1, cartilage-derivedmor-phogeneticprotein-1; ROS, reactive oxygen species; BMSCs, bone marrow mesenchymal stem cells.

Here, we constructed a nanoparticle with low toxicity, ROS response properties, and ability to eliminate joint inflammation and induce cartilage repair, which was named as DLNPs. And we used hydrogen peroxide (H_2_O_2_) as a positive control. The nanoparticles use -SeSe- group as the ROS response component and DEX and CDMP-1 as the main pharmacophore. Drug-carrying nanoparticles were directly delivered to joint lesions through joint cavity injection. By taking advantage of the high concentration of oxygen free radicals in arthritis lesions, the fracture of -SeSe- and the slow release of DEX and CDMP-1 were observed. At the same time, PLGA hydrophobic groups accumulated in the damaged cartilage due to hydrophobic effect, forming exogenous biological scaffold. The schematic diagram of the nanoparticle for the treatment of osteoarthritis was shown in Scheme 1.

## Materials and Methods

### Experimental Materials

#### Reagents

Dexamethasone, poly (lactic-co-glycolic acid (PLGA), mPEG_5k_-COOH, selenocystamine, N-(3-dimethylaminopropyl)–n′-ethyl carbondiimine hydrochloride (EDC), n-hydroxysuccinimide (NHS), and N, N-diisopropylamine (DIPEA) were obtained from Energy Chemical. Chloroform, polyvinyl alcohol (PVA) and D-α-tocopherol polyethylene glycol succinate (TPGS) were obtained from Alladin.

Lipopolysaccharides (LPS), rhodamine-B (RhB), penicillin, streptomycin, and 4′, 6-diamidino-2-phenylindole (DAPI) were also purchased from Aladdin. Trypsin and fetal bovine serum (FBS) were obtained from Gibco. CDMP-1 antibody (Product No. R30403) was obtained from Yuanye Bio-Technology.

#### Instruments

The techniques used in this study included high-performance liquid chromatography (HPLC, Dionex UltiMate 3000, ThermoFisher), transmission electron microscopy (TEM, Hitachi, H-600), fluorescence microscopy (Zeiss 710, Jena, Germany), dynamic light scattering (DLS, Santa Barbara, Nicomp 380 ZLS), laser scanning confocal microscopy (Zeiss 710, Germany) and flow cytometry (BD Biosciences, BD FACS Canto II).

#### Cells and Animals

Macrophages (RAW 264.7) were purchased from BeNa culture Collection. New Zealand rabbits were purchased from the Model Animal Research Center of Nanjing University.

Animal experiments were performed according to protocols approved by the Ethical Committee of XuZhou Central Hospital.

### Methods

**Figure 1:**
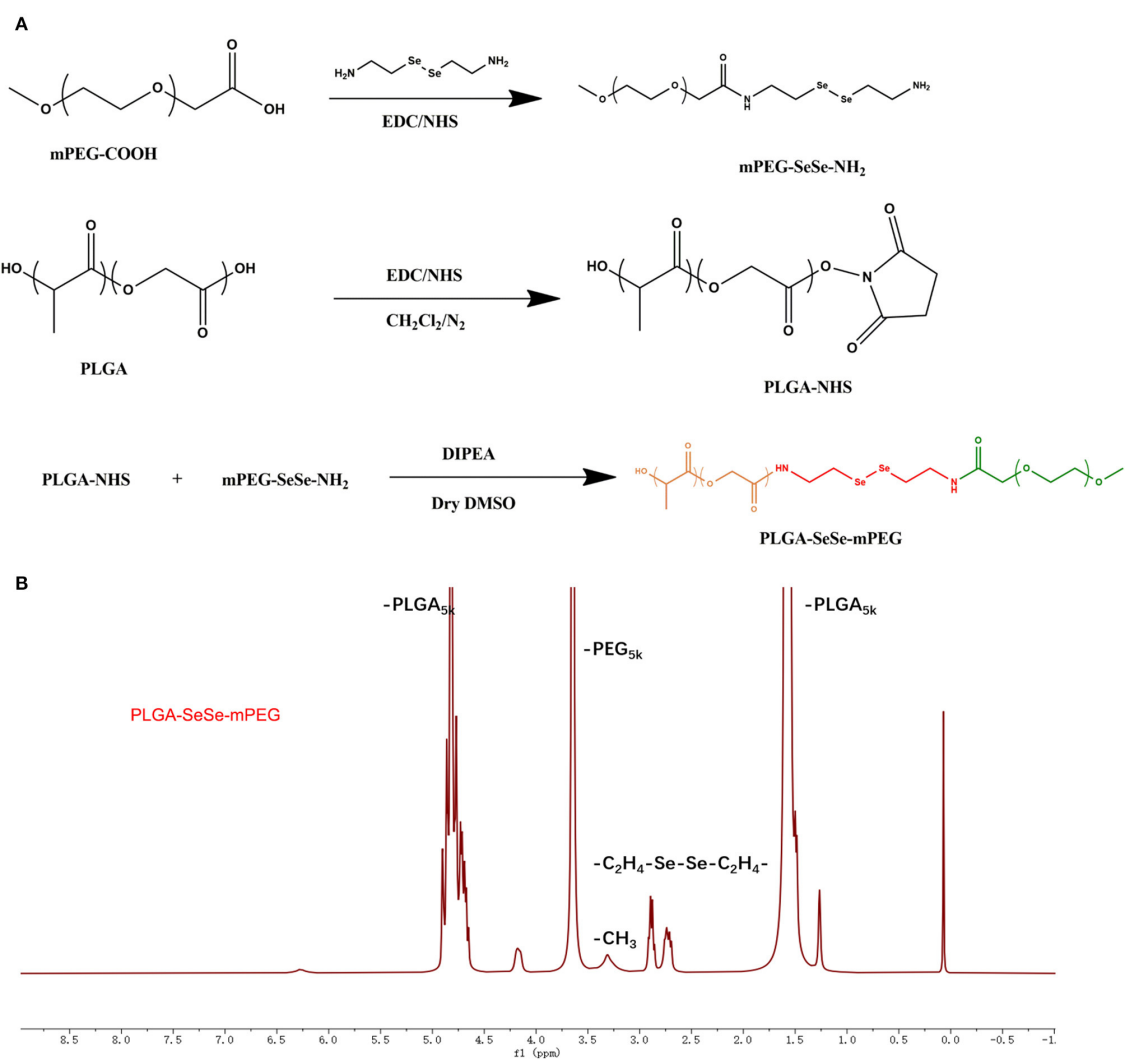
Schematic diagram for synthesis **(A)** and Nuclear magnetic resonance spectroscopy **(B)** of PLGA-SeSe-Mpeg. PLGA, poly(lactic-co-glycolic acid); PEG, polyethylene glycol.

The PLGA-SeSe-mPEG synthesis process was depicted in Figure 1A. The nuclear magnetic resonance data of the PLGA-SeSe-mPEG was present in Figure 1B.

#### Preparation of mPEG-SeSe-NH_2_

The mPEG_5k_-COOH was dissolved in formamide with EDC and NHS (mPEG_5k_-COOH: EDC: NHS = 1:5:5). The mixture was stirred for 6 h to mix it thoroughly and then a mixture of selenocystamine and formamide (V/V = 1:9) was dripped at 0°C and allowed to react for another 5 h. Excess precooled acetone was added and allowed to crystallize into crystals, which were filtered through a microporous membrane (0.22 mm). The precipitate products were then dissolved in water and dialysed (MWCO: 3,500) for 48 h. The intermediate product, mPEG-SeSe-NH_2_, was obtained by freeze-drying.

#### Synthesis of PLGA-NHS

PLGA (0.65 g), EDC (80 mg), and NHS (48 mg) was dissolved in 10 mL of dichloromethane, and reacted for 24 h under nitrogen protection. The product was then precipitated by precooled aether and the residue NHS and EDC were removed by washing three times with a mixture of aether/methanol (50/50, v/v). The resulting sediment was vacuum-dried and labeled PLGA-NHS.

#### Preparation of the PLGA-SeSe-mPEG

Briefly, PLGA-NHS (0.1 g), mPEG-SeSe-NH_2_ (0.2 g), and DIPEA (6 uL) were dissolved in a dry solution of dimethyl sulfoxide. The solution was stirred under nitrogen protection for 48 h. The solution was dialysed (MWCO: 8,000–14,000) for 48 h, and the final product PLGA-SeSe-mPEG was obtained by freeze-drying.

#### Assembly of Blank Particles

PLGA-SeSe-mPEG (50 mg) was added to 1 mL of chloroform and mixed into 6 mL of a 1% PVA and TPGS mixture (PVA: TPGS 1:5). An 80 W ultrasound probe was used for 2 min in an ice bath to form an oil-water emulsion.

The emulsion was then added to 30 mL of 0.3% PVA and stirred overnight to solidify the surface. Ultrafiltration concentration was carried out with a 100 KD ultrafiltration tube. Finally, the volume of the mixture was fixed with pure water to 5 mL, and the samples were collected and stored at 4°C.

#### Assembly of Drugs-Loaded Particles Containing DEX and CDMP-1

DEX, CDMP-1, and PLGA-SeSe-mPEG were dissolved in trichloromethane, respectively. Next, 50 μL of 2 mg/mL DEX, 50 μL of 1 mg/mL CDMP-1, 50 μL of 1 mg/mL rhB, and 500 μg of the PLGA-SeSe-mPEG mixture were evenly and ultrasonically prepared for use. The process for producing drug-loaded particles was the same as that of blank particles described above.

### Characterization of Micellar Particles

The size and potential of the nanoparticles were measured by DLS, and the morphology of the nanoparticles was characterized by TEM (120 keV, 5,000–30,000×).

To prepare samples for morphological characterization, a total of 5 μL of the formulation was deposited onto a carbon-disc ultrathin grid, followed by staining with 0.2% phosphotungstic acid for 30 s.

A total of 0.1 mM H_2_O_2_solution was used to simulate the ROS environment at the inflammatory lesions.

### Drug-Loading Coefficient and Drug Release

Drug-loaded nanoparticles (5 mL) were transferred into a dialysis bag (MWCO, 12–14 K) and submerged into 45 mL of deionised (DI) water (37°C, pH 7.4). At set time points (10 min, 30 min, 1 h, 1.5 h, 2 h, 3 h, 4 h, 6 h, 8 h, 12 h, 24 h, 48 h, and 72 h), 1 mL of dialysate was taken as a sample and 1 mL of fresh DI water was refilled. After the samples were separated by HPLC, the DEX contents were determined by mass spectrometry, and the contents of CDMP-1 were determined by an enzyme-linked immunosorbent assay (ELISA) kit.

A total of 0.1 mM H_2_O_2_ solution was used to replace the DI water to prepare dialysate to test the ROS sensitivity of drug-loaded nanoparticles. The rest of the procedure was the same as the control group.

### Isolation and Culture of BMSCs

BMSCs cells were isolated by density-gradient centrifugation.

After anesthesia was successfully performed on healthy 1-month-old New Zealand rabbits, the skin was prepared and lower limbs were disinfected with 75% alcohol and 3% iodine. Under aseptic conditions, 5 mL of bilateral iliac spine bone marrow was rapidly extracted by a puncture with a 20 mL syringe. The same amount of Dulbecco's modified Eagle medium without FBS was extracted with a sterile 5 mL syringe on an ultra-clean table, and the cells were resuspended. Then the cells were then placed into a screw centrifuge tube, centrifuged at 800 rpm for 6 min, and the supernatant was discarded to remove tissue cells, such as fat and periosteum. The cells were resuspended in a complete cell culture medium containing 10% FBS, and then slowly injected into a centrifuge tube containing a 10 mL of lymphoid separation fluid at a volume ratio of 1:1. After centrifuging at 1,500 rpm for 30 min, the annular pale-yellow monocyte cloud layer at the junction of the liquid level was taken and resuspended in a complete cell culture solution to make a single-cell suspension, which was then counted with a blood-count board under an optical microscope. The suspension was then inoculated with a density of 2 × 10^5^/mL in a 25 cm^2^ plastic culture bottle and placed in a constant-temperature incubator at 37°C in 5% CO_2_ at saturated humidity. After most of the cells grew adherent and slightly shook the bottle, the non-adherent cells were discarded and labeled as primary cells.

After ~12–14 days of primary cell culture, the cells were filled with culture bottles. At this time, they exhibited a fibroblast-like appearance.

Finally, cell passage cultivation of BMSCs were cultured at a 1:3 ratio every 8 days, and the non-adherent cells were discarded in each exchange to purify BMSCs. P3-generation BMSCs with good growth status were selected as experimental cells.

### Activation of Macrophages

RAW 264.7 cells were activated by incubating them in a complete medium containing 0.5 μg/mL LPS for 12 h.

### Cell Proliferation

The BMSCs and the activated macrophages (1 × 10^4^ cells/well) were treated with various concentrations of PLGA-SeSe-mPEG micelle and DEX&CDMP-1@PLGA-SeSe-mPEG micelle for 1, 3, and 7 days. Cell viability was determined using an MTT assay.

### Histochemical Detection of Collagen Type II

BMSCs induced for 21 days by CDMP-1 and drug-loaded micelles were digested into a cell suspension, and then fixed with 4% paraformaldehyde at 4°C for 30 min. The membrane was broken with 0.1% Triton solution for 30 min, sealed at room temperature for 30 min, and then cleaned with PBS solution 3 times.

Polyclonal antibodies against type II collagen were diluted at a ratio of 1:100, incubated overnight at 4°C and an SABC immuno-histochemical staining kit was used following the kit instructions. Finally, a DAB kit was used to detect the expression of type II collagen in the tissues, with a positive reaction producing a brown-yellow color. PBS was used for negative control staining instead of an antibody.

### Alsin Blue Stain

BMSCs were inoculated in 12-well plates with 5,000 cells/well and cultured for 21 days in an induction solution containing 100 ng/mL drug-loaded micelles. BMSCs were rinsed with PBS for 3 min and fixed with 4% paraformaldehyde for 30 min. The cells were then rinsed with 0.1 mol/L of HCl for 5 min. After the pH was lowered to 1, a 0.1% alsin blue solution was used to stain the BMSCs overnight. The samples were then stained with a 0.1% neutral solid red solution for 5 min, rinsed with distilled water and dried, sealed with neutral resin and placed under an inverted microscope for imaging.

### Cellular Uptake *In vitro*

The intracellular localization of drug-loaded nanoparticles was further detected by confocal imaging. BMSCs and activated macrophages were cultured in glass-bottom dishes at a density of 2 × 10^5^ cells/well for 24 h. After 2 h of incubation with drug-loaded nanoparticles (10 μg/mL), the cells were washed with a PBS buffer three times

The cells were then fixed with 4% (v/v) paraformaldehyde and stained with Hoechst for 15 min before observation with a laser scanning confocal microscope.

Intracellular uptake of drug-loaded nanoparticles was detected by flow cytometry.

### Expression of Key Proteins Measured by ELISA

Activated macrophages and BMSCs were planted in 6-well plates at a density of 2 × 10^5^ cells/well and cultured for 24 h. The cells were further treated by different concentrations of nanoparticles for 24 h.

After the culture medium in the culture plate was removed, the cells were digested with trypsin, and an appropriate amount of culture medium was added to wash the cells off the culture plate.

The cell suspension was collected into a centrifuge tube, centrifuged at 1,000 *g* for 10 min, and then the culture medium was removed and the cells were washed three times with precooled PBS.

Cells were resuspended by adding 150–250 mL of PBS to each well of the 6-well plates and then lysed by ultrasonic treatment of the suspension with an ultrasonic cell breaker.

At 4°C, the ultrasound-treated cell fluid was centrifuged at 10,000 *g* for 10 min to remove cell debris, and the supernatant was then collected and labeled.

The cell supernatants were further analyzed by ELISA to determine the concentration tumor necrosis factor alpha (TNF-α), interleukin-1beta (IL-1β) and type II collagen.

### Statistical Methods

SPSS13.0 statistical software package was used for analysis. Data were expressed as mean ± standard deviation. One-way ANOVA was used for comparison between groups with *P*-value & LT 0.05 was considered statistically significant.

## Results and Discussion

The synthesis path of the polymer PLGA-SeSe-mPEG was prepared as shown in Figure 1A. The nuclear magnetic resonance spectroscopy of PLGA-SeSe-mPEG (Figure 1B) showed that in addition to the characteristic peaks of -PLGA and -mPEG, there was an obvious characteristic peak of -SeSe- at 2.6–2.9 ppm.

**Figure 2:**
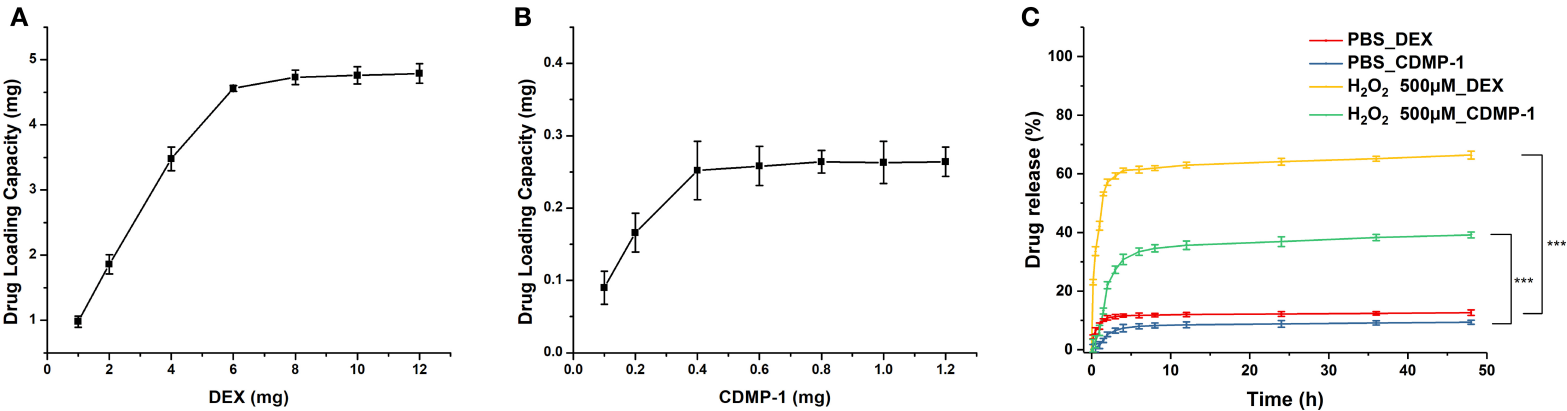
Size distribution of the nanoparticles after treatment with different concentrations of H_2_O_2_ characterized by DLS. Morphological characterization of PLGA-SeSe-mPEG micelle **(C)** and uncoated nanoparticles **(D)**. DLS, dynamic light scattering.

As shown in the DLS results (Figure 2A), the particle-size distribution of blank and drug-loaded nanoparticles displayed an obvious single-peak normal distribution, without trailing tail or double peak, which indicated that the sizes of the micelles were relatively uniform. The drug load significantly increased the size of nanoparticles from 260 to 295 nm with a wider size distribution.

After removing the mPEG shell by stimulation with H_2_O_2_, the size distribution of the nanoparticles showed a multi-peak distribution (Figure 2B). As the concentration of H_2_O_2_ increased, the proportion of secondary peaks on both sides of the main peak increased. When the concentration of H_2_O_2_ reached 500 μM, the micelle particles were completely destroyed, and the particle size presented an obvious bimorphic distribution, indicating an obvious aggregation effect of nanoparticles after uncoating.

As shown in the TEM results (Figures 2C,D), blank micelles presented an obvious core-shell structure with a relatively light hydrophilic ringing around the hydrophobic core. Blank micelles embraced a relatively uniform particle size with significant particle spacing and clean particle backgrounds. However, after stimulation by H_2_O_2_, the hydrophilic shell of the nanoparticles fell off, and accumulation of the hydrophobic nanoparticles was evident, resulting in different particle sizes. The detached mPEG segments were dispersed in the solution, contributing to a dirty background for the nanoparticles.

Previous studies have shown that the most important factor in determining drug loading is the compatibility between the hydrophobic segment and the drug molecule (Shi et al., 2016; Wilkosz et al., 2018). Factors such as the amount of drug loaded, particle size and particle-size distribution vary by drug-loading method. Exploring suitable drug-loading methods that enable polymer nanoparticles to carry larger quantities of drugs is the main content of this section (Zhang et al., 2017).

Drug-loading performance tests of blank micelles were conducted by assembling drugs of different concentrations with 50 mg blank micelles. The prepared drug-loading nanoparticles were filtered out of the unwrapped drugs by dialysis, and then dissolved into the organic solvent again to detect the drug content in the organic solvent, and to predict the drug-loading amount.

**Figure 3:**
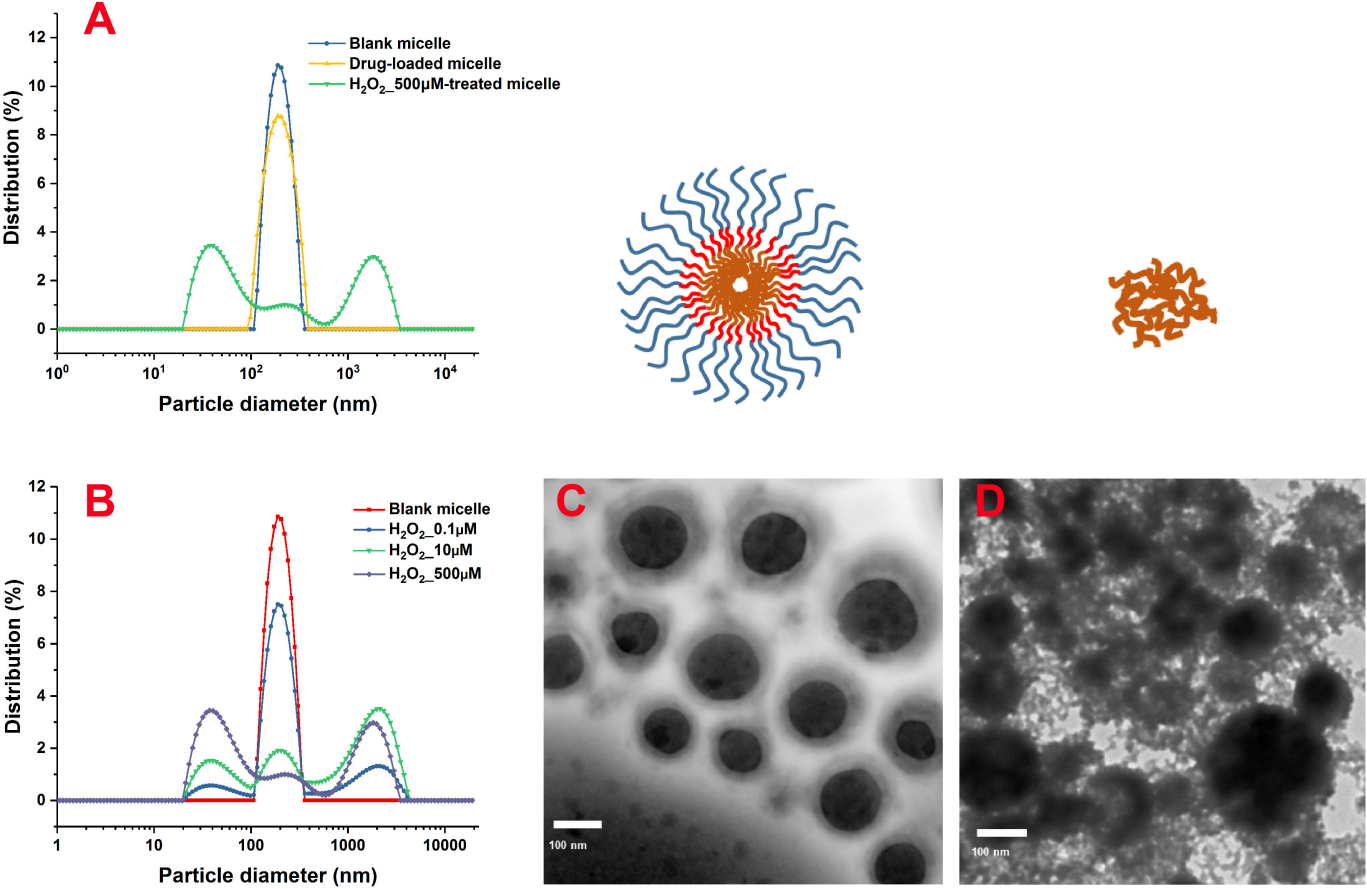
The DEX **(A)** and CDMP-1 **(B)** loading performance of blank micelles, and accumulative DEX and CDMP-1 release with or without ROS_500μ*m*_ **(C)**. DEX, dexamethasone; CDMP-1, Cartilage-derivedmor-phogeneticprotein-1; ROS, reactive oxygen species. All error bars were presented as mean ± SD. ^***^p < 0.001.

Drug-loading results (Figures 3A,B) showed that a 50 mg blank micelle could load a maximum of 4.8 mg of DEX and 0.265 mg of CDMP-1, with drug-loading rates of 9.8 and 0.53%, respectively. A PBS buffer (pH 7.4) was used as the release medium. The drug-loaded nanoparticles prepared by the physical-embedding method generally released the drug through diffusion, which was faster than the drug-loaded nanoparticles prepared by chemical binding. Its release rate was related to factors such as the molecular weight of the nanoparticles and the interaction between the drug and the nanoparticles. Strong compatibility between the drug and the hydrophobic core can delay the release of the drug, and a strong hydrogen bond between the micellar core and the drug can also slow down the release of the drug.

As shown in Figure 3C, DEX and CDMP-1 both had drug-release rates of <10% in PBS. When the concentration of H_2_O_2_ reached 500 μM, the release rate of DEX reached more than 60% within 4 h, while the release rate of CDMP-1 was slightly lower, at 37.7% of CDMP-1. Because amphiphilic biomaterial is sensitive to H_2_O_2_, the concentration of hydrogen peroxide can be reduced at the ischaemic site, limiting damage to the body. The H_2_O_2_-sensitive amphiphilic biomaterial of the present invention is used as a drug carrier to meet the needs of extended circulation and targeted therapy (Shah et al., 2020).

**Figure 4:**
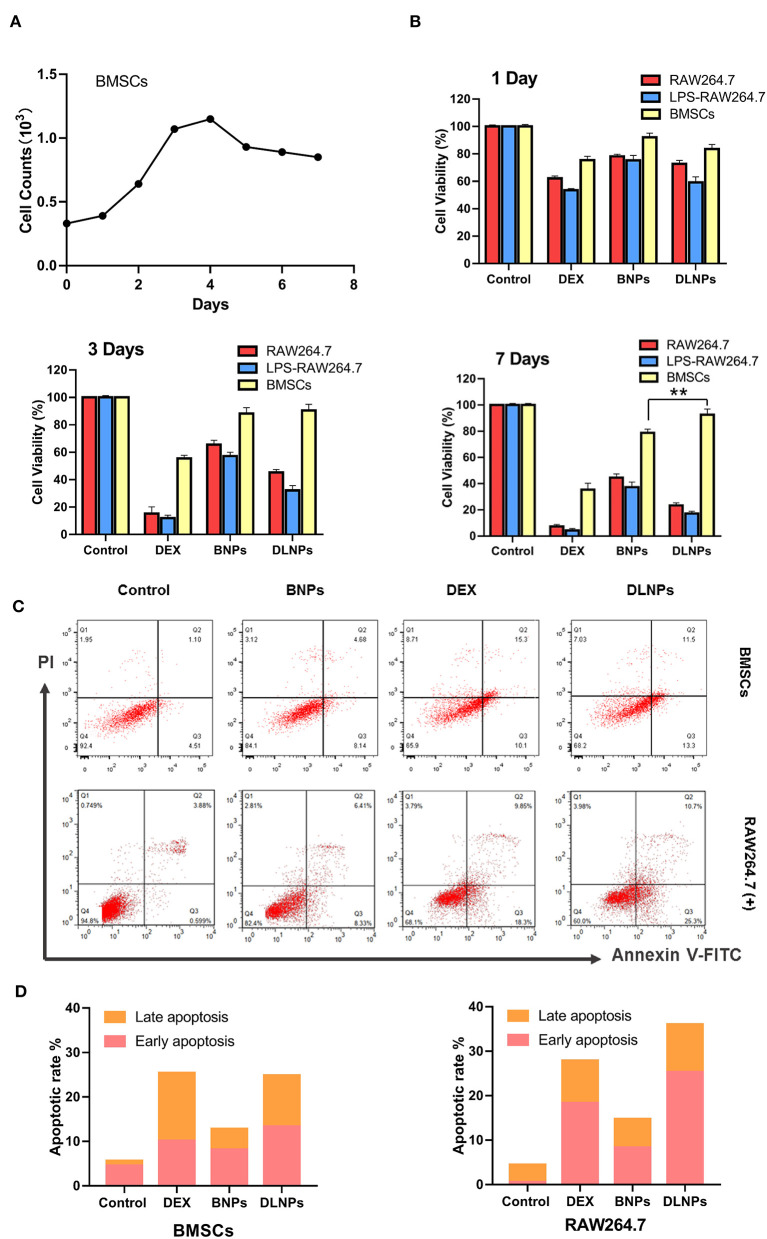
Cell counts of BMSCs Cells for 7 days **(A)**.Cell viability of RAW 264.7 and BMSCs cell lines after 1 day, 3 days, and 7 days incubation in response to various treatments **(B)**. Flow cytometric analyses **(C)** and quantitative analysis **(D)** of activated RAW264.7 and BMSCs after 4 h of incubation with various treatments, with PBS serving as the control. All error bars were presented as mean ± SD. ^**^p < 0.01. BMSCs, bone marrow mesenchymal stem cells; PBS, phosphate buffer saline.

In order to verify the stem cell characteristics of extracted BMSCs, BMSCs were incubated for a long time and the cells were counted. As shown in Figure 4A, BMSCs entered the logarithmic growth phase at 2–4 days, and the number of cells decreased significantly and entered the plateau phase at 5 days, indicating that BMSCs also had significant contact inhibition effect.

The cytotoxic results of drug-loaded micelles and DEX were shown in Figure 4B. The addition of DEX significantly reduced the activity of macrophages, but had little effect on the activity of BMSCs. Blank nanoparticles were less cytotoxic to all three groups of cells, with cell survival rates exceeding 80%, indicating the potential of blank nanoparticles to serve as drug carriers. Drug-loaded nanoparticles exhibited cytotoxicity to certain macrophages. When treated with drug-loaded nanoparticles, macrophages exhibited a cell survival rate of approximately 73%, and the survival rate of activated macrophages was even lower, at 57%. Longer incubation resulted in a decrease in the relative number of treated macrophages. However, blank micelles and drug-loaded micelles had little effect on BMSCs. After 7 days of culture, BMSCs treated with DLNPs had higher activity than those treated with BNPs. Combined with the proliferation curve of BMSC, DLNPs treatment reduced the contact inhibition effect and restored the vitality of BMSCs.

Measurement of flow-cell apoptosis (Figures 4C,D) showed that DEX significantly affected the induction of apoptosis of macrophages, with the cell survival rate dropping from 92.4 to 65.9%. DEX had a relatively light effect on BMSCs, with the cell survival rate dropping from 87.2 to 77.5%. Blank nanoparticles had little effect on apoptosis induction in the two groups; the proportion of normal cells was more than 80%, indicating that blank nanoparticles were safe and non-toxic. Drug-loaded nanoparticles showed a similar apoptotic effect with DEX on activated macrophages, with the survival rate dropping to 68.2%. Drug-loaded nanoparticles had little effect on BMSCs, up to 75% of normal cells.

The fluorescent dye RhB was attached to the nanoparticles to indicate the location of the drug-loaded micelle inside the cell after being engulfed.

**Figure 5:**
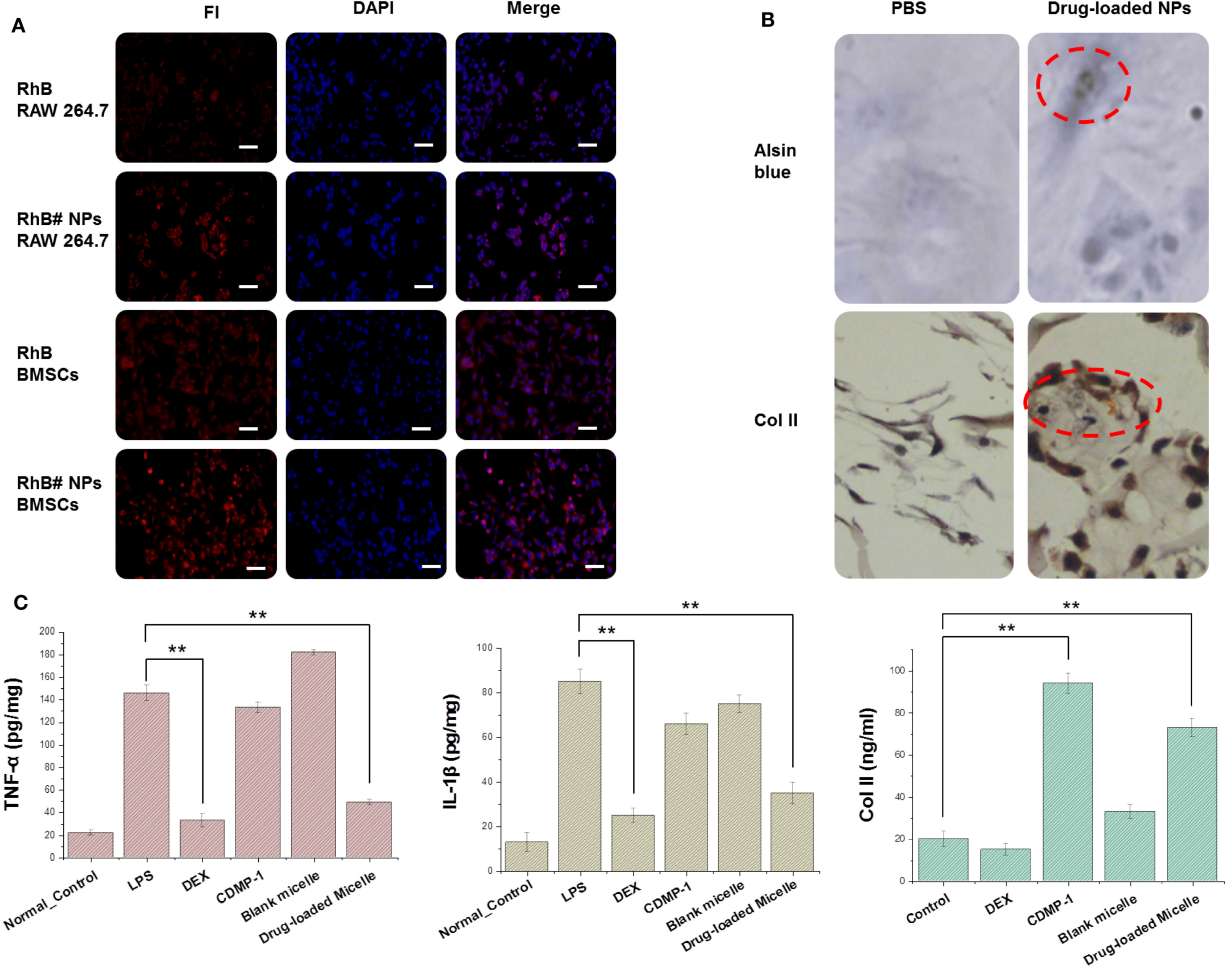
**(A)** Cellular uptake of RhB and RhB#Nanoparticles in activated RAW 264.7 and BMSCs while the nucleus were stained with DAPI. **(B)** Results of chondrogenic differentiation of BMSCs were conducted by type II collagen immunohistochemistry staining and Alsin blue staining. **(C)** Expression of key factors (TNF-α, IL-1β, and type II collagen) were tested by an ELISA kit. All error bars were presented as mean ± SD. ^**^p < 0.01. ELISA, enzyme-linked immunosorbent assay; TNF, tumor necrosis factor; IL, interleukin.

Cytophagocytosis diagrams (Figure 5A) showed that the drug-loaded nanoparticles accumulated primarily in the cytoplasm and did not enter the nucleus. The differences in cell phagocytosis between cells were significant. The activated macrophages were the brightest, followed by the inactive macrophages and the BMSC group.

After incubating CDMP-1 and drug-loaded micelles with BMSCs for 14 days, BMSCs were fully induced to form cartilage. Collagen and chondrogenic differentiation in cells was characterized by the histochemistry of alsin blue and type II collagen. As shown in Figure 5B, after 2-day differentiation induced by drug-loaded nanoparticles, a small number of BMSCs changed from a spindle shape to polygons, and the black particles around the nucleus gradually increased with induction time. After 14 days of differentiation induction, the cells began to grow in size, particles around the nucleus were visible, and the cells gradually grew into clusters of cartilaginous nodules. Type II collagen immunohistochemistry staining showed that some cells were dark brown and exhibited a strong positive reaction. While the positive signals were located mainly in the cytoplasm of the cells, a small number of positive signals occurred in the extracellular matrix, indicating that the cells expressed type II collagen after the induced differentiation.

CDMP-1 and drug-loaded nanoparticles were incubated with BMSCs for 24 h, and the supernatant contents of TNF-α, IL-1β, and type II collagen were determined by ELISA, as shown in the Figure 5C. The expression of TNF-α and IL-1β in activated macrophages was significantly increased, compared with normal macrophages. DEX and drug-loaded nanoparticles therefore significantly reduced the expression level of the above factors, indicating that DEX and drug-loaded nanoparticles had a significant inhibitory effect on activated macrophages and reduced inflammatory responses caused by LPS. Both CDMP-1 and drug-loaded nanoparticles significantly increased the expression level of type II collagen, indicating that CDMP-1 and drug-loaded nanoparticles effectively induced chondrogenic differentiation of BMSCs. All groups showed statistically significant differences (*P* < 0.05). This indicated that, at a higher concentration, the action time was longer, which caused drugs that entered the cell through either of two ways to act fully on the cell and perform its function.

## Conclusion

OA is usually associated with cartilage damage and joint inflammation (Chow and Chin, 2020). We designed, synthesized, and assembled a core-shell nanomicelle loaded with the anti-inflammation drug DEX and CDMP-1 to induce BMSCs to repair cartilage and inhibit joint inflammation. The nanometre-scale micelles were able to release drugs at lesion sites with high ROS, making them ROS-sensitive.

The function of the nanometre-scale micelle drug was verified through drug release, cytotoxicity, cartilage-induced differentiation, and other experiments, providing a basis for the further development of the drug in the treatment of OA.

## Data Availability Statement

The raw data supporting the conclusions of this article will be made available by the authors, without undue reservation.

## Author Contributions

XW, WH, and CH: conception and design. CH, BL, and QX: administrative support. XW and WH: provision of study materials. JC, PL, and QX: collection and assembly of data. XW, PL, and CH: data analysis and interpretation. All authors: manuscript writing and final approval of manuscript.

## Conflict of Interest

The authors declare that the research was conducted in the absence of any commercial or financial relationships that could be construed as a potential conflict of interest.

## Abbreviations

OA: Osteoarthritis
DEX: dexamethasone
CDMP-1: cartilage-derivedmor-phogeneticprotein-1
Hydrogen peroxide, ROS: reactive oxygen species
BMSCs: bone marrow mesenchymal stem cells
PLGA: poly(lactic-co-glycolic acid
PEG: polyethylene glycol
DLS: dynamic light scattering
PBS: phosphate buffer saline
ELISA: enzyme-linked immunosorbent assay
TNF: tumor necrosis factor
IL: interleukin.
